# A Biopsychosocial Model of Mealtime Management in Persons with Dementia, an Asset-Based Approach to Patient-Centered Care

**DOI:** 10.3390/geriatrics7050112

**Published:** 2022-10-07

**Authors:** David F. Bayne, Samantha E. Shune

**Affiliations:** College of Education, University of Oregon, Eugene, OR 97403, USA

**Keywords:** dementia, dysphagia, mealtime, management, model, intervention, theory

## Abstract

Considering the rapid increase in the population over the age of 65, there is increasing need to consider models of care for persons with dementia (PWD). One common deficit associated with dementia progression is difficulty with successful participation in mealtimes. Difficulty participating in mealtimes in PWD is not the result of one factor, but rather a confluence of biological, psychological, and social characteristics common in dementia. Factors leading to mealtime difficulties for PWD may include changes in cognitive status, altered sensorimotor functioning, and increased reliance on caregiver support. The complex nature of biological, psychological, and social factors leading to mealtime difficulty highlights the need for a pragmatic model that caregivers can utilize to successfully support PWD during mealtimes. Existing models of dementia and mealtime management were reviewed and collated to create a model of mealtime management that considers this complex interplay. The Biopsychosocial Model of Mealtime Management builds on past research around patient-centered care and introduces an asset-based approach to capitalize on a PWD’s retained capabilities as opposed to compensating for disabilities associated with dementia. We hope this model will provide a framework for caregivers to understand what factors impact mealtime participation in PWD and provide appropriate means on intervention.

## 1. Introduction

In 2017, 15.6% of the American population was 65 years or older, representing more than 1:7 Americans [1]. By the year 2060, the number of individuals in America over the age of 65 is expected to double [2]. An increasingly aging population generally reflects positive developments in healthcare leading to increased life-expectancy; however, this is accompanied by inevitable increases in biological and neurological decline seen in aging bodies. The natural degenerative aging process is the largest contributing factor to the development of non-communicable diseases [3].

Dementia, one of the most prevalent non-communicable diseases, is a term used to describe a cluster of symptoms that results from various neurodegenerative diseases. There are over 100 types of dementia, most commonly presenting as Alzheimer’s disease, vascular dementia, Lewy body dementia, and Frontotemporal dementia [4]. Worldwide, the number of persons with dementia (PWD) is estimated to be 47 million, a number expected to increase to 131 million by the year 2050 [5]. Cognitive decline is not the only symptom associated with a dementia diagnosis; behavioral and psychiatric symptoms (BPSD) oftentimes fall into the cluster of symptoms that hallmark the neurodegenerative disorder [6]. Thus, in order to maximize health and quality of life within this growing population, it may be crucial to consider both the biological as well as the psychosocial deficits when implementing a holistic approach to management of dementia.

One primary area of management in dementia care relates to nutritional intake, which is often complicated by the presence of oropharyngeal dysphagia (OD). The prevalence of OD varies greatly across settings, but up to 70% of all referrals for dysphagia assessment are for older adults [7]. OD is commonly seen in PWD, with 57–93% experiencing swallowing-related deficits [8,9]. Given the number of individuals expected to develop dementia, these statistics represent a potential 26.8 million individuals with dementia who will experience some degree of swallowing-related deficit. Yet, despite the high potential of OD in PWD, a consensus on the functional impact of swallowing-related deficits in this population is lacking.

OD among PWD likely results from many different factors that accompany the biological, cognitive, and psychosocial decline during the disease’s progression. OD results from disturbances in motor control, changes in cognition, and/or sensory issues, all of which are common in individuals with progressive neurological diseases [10]. The physiological causes of OD in PWD are varied, but likely related to age-related decline in motor and sensory functioning that is exacerbated by the progressive neurological decline characteristic of dementia [11]. Unfortunately, the consequences of OD can be severe. Even in otherwise healthy older adults, aging cognitive and neuromuscular processes increase the risk for malnutrition and the development of aspiration pneumonia, which puts these individuals at higher risk of mortality [12,13]. In addition to the physiological effects of dysphagia, including malnutrition, weight loss, and dehydration, OD can impact psychosocial domains, resulting in reduced social participation and quality of life (QoL) [12,14,15,16]. PWD, in particular, face a wide range of symptomology congruent with the deleterious effects of OD, including reduced sensory and motor function resulting in altered feeding ability [8,10], which may further reduce social participation and quality of life in PWD.

## 2. Current Models That Can Inform Mealtime Management

Prior to considering plans for mealtime management in PWD, it is crucial to consider what is known about mealtime management in both clinical and non-clinical populations. A number of current theoretical models of mealtime management exist that can help inform a new conceptualization of mealtime management of PWD. Three models will be discussed below, The World Health Organization’s International Classification of Function, Disability, and Health, Bisogni et al.’s Framework of Typical Mealtime Processes, and historical perspectives on the Biopsychosocial Model of Patient care. Each model presents with both strengths and limitations when considering successful mealtime management for PWD, as will be described further below. Building on the unique strengths of each, these three theoretical models were all used to inform the Biopsychosocial Model of Mealtime Management in PWD proposed here.

### 2.1. World Health Organization’s International Classification of Function, Disability, and Health

The World Health Organization’s International Classification of Function, Disability, and Health (WHO-ICF) offers a conceptual model for describing an individual’s functional status within the context of disease or disorder [17]. Approved by all member states of the World Health Organization, the WHO-ICF model is one of the most widely accepted models for classification of disfunction within the context of health. The health condition (a disease or disorder) may impact functioning at three mutually interacting levels: in relation to the body, activity, and participatory capability within the context of an individual’s environmental and personal factors [17]. Significantly, the model explicitly recognizes, and draws attention to, the important role of contextual factors on functional outcomes given the presence of disease.

As applied to mealtimes in dementia, the relationship between dementia (disease) and the eating process (activity) is multifaceted. Successful or unsuccessful mealtimes cannot be attributed to any one factor and, as the WHO-ICF model stipulates, multiple contextual factors, such as reliance on others for feeding assistance and altered cognitive functioning, affect the functional outcome. However, it must be recognized that this model of classification is born out of the identification of disability within the context of the person’s diagnosis and environment as opposed to an individual’s preserved abilities. The 2013 WHO Practical Manual for using the ICF framework mentions the word “disability” 270 times, and mentions the word “enable” in the context of human performance twice; the word “ability” is mentioned in the same context just once [18]. This deficit-based terminology may contribute to furthered disease-based treatment approaches that respond to a patient’s disfunction as opposed to a patient-centered approach which leverages the patient’s retained abilities for mealtime success. Combining the WHO-ICF, which highlights the important interconnected nature of disease, function, and environment, with a more asset-based approach to dementia management, which aims to leverage those retained abilities, may better encourage patient-centered care that frames PWD and caregivers as co-producers of positive health outcomes [19].

### 2.2. Bisogni et al.’s Framework of Typical Mealtime Processes

**Figure 1:**
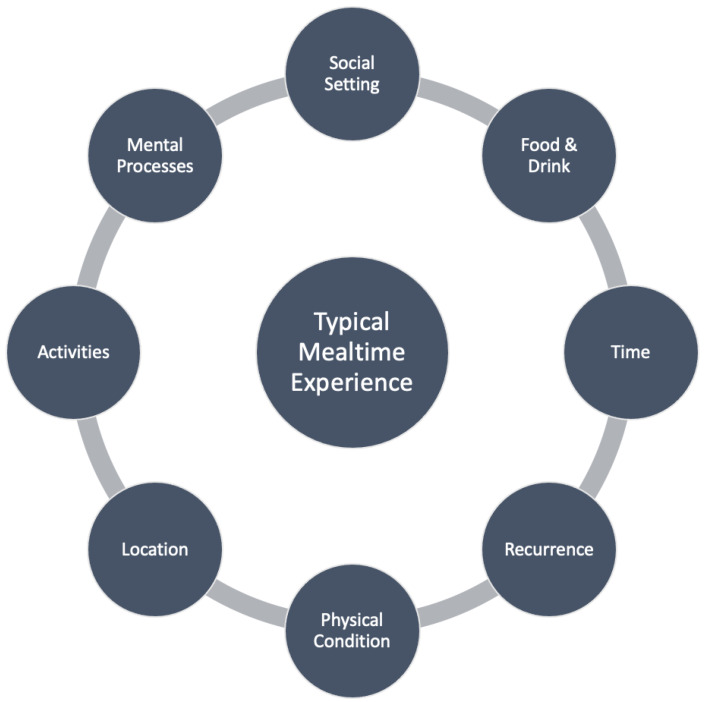
The eight interacting dimensions and features of eating and drinking episodes that characterize situational food and beverage consumption among working adults (based on [20]).

Moving toward a more asset-based approach to mealtime management for PWD requires a clear understanding of the environmental factors related to mealtimes. A 2007 review of eating habits in healthy adults revealed an intricate series of interconnected dimensions that form a framework to describe the typical mealtime process [20]. These dimensions include social setting, food and drink, time, recurrence, physical condition, location, activities, and mental processes (see Figure 1). Social setting describes the people present and their relationship to the participant. The food and drink domain details the type of material consumed, amount consumed, and how the food or drink was prepared (e.g., homemade or pre-prepared). The dimension of time is described as the time of day the meal was consumed, the chronological relation to other daily experiences (e.g., after exercise or before work), and the subjective experience of time (e.g., participants reported “I was in a rush”). The domain of recurrence was used to describe how repetitious the mealtime experience was (e.g., a meal eaten once a week versus once a year on special occasions). Physical condition refers to two main components, the appetite and hydration needs of the participant and the physical state of the participant such as presence of fatigue, illness, or disease. The location domain describes both the general location (e.g., at home vs. at a restaurant) as well as positionality within that location (e.g., at the dining room table versus in front of the television). Activities include anything that was happening during the mealtime (e.g., parental tasks) and how disruptive they were to the mealtime experience. Lastly, the mental processes domain includes two main features, food-related goals (e.g., eating so the food does not go bad) and associated emotions (e.g., stressed versus at ease).

Within Bisogni et al.’s (2007) conceptual model, these dimensions come together to characterize a mealtime episode with each dimension describing a particular aspect of the mealtime. For example, mapping a typical dinnertime using this framework may include location (at home), people (with family), and time (after work and picking up children); each of these aspects color the eating experience and converge to define its success or failure. Mapping the mealtime experience of a PWD in a long-term care (LTC) facility may include location (in an isolated room), social setting (alone, without peers), and mental processes (confusion, frustration). Each of these dimensions influences the others, and the interconnected nature of these dimensions affects the ultimate mealtime experience.

### 2.3. Historical Perspectives of the Biopsychosocial Model of Patient Care

Bisogni et al.’s (2007) description of the mealtime experience as a dynamic process of environmental–social–personal interactions supports a more holistic approach to patient care, which is often framed within a biopsychosocial model. The biopsychosocial model of patient care was introduced as an alternative to the biomedical model of illness classification [21]. Engel’s Biopsychosocial model argued that a patient’s biological, social, psychological, and behavioral domains must be considered together in order to fully understand a patient’s diagnosis and prognosis [21]. Adaptation of the biopsychosocial model to describe dementia was first by described by Cohen-Mansfield, who proposed that the manifestation of dementia is the result of the convergence of biological, psychological, and environmental factors [22].

Considering the progressive nature of dementia, Spector and Orrell (2010) expanded on Cohen-Mansfield’s (2000) model by proposing that the impact of biological, psychological, social, and environmental domains change throughout disease progression [23]. In addition to highlighting the progressive nature of dementia-related symptomatology, the Spector-Orrell model identifies fixed and tractable biopsychosocial characteristics that a PWD experiences. Fixed characteristics are those that are not able to be altered. For example, fixed biological characteristics may be diagnosis or past medical history and fixed psychosocial characteristics may be personality traits or previous life events. Tractable characteristics are those that can be changed. For example, a tractable biological characteristic may be sensory impairment, and a tractable psychosocial characteristic may be environment or levels of mental stimulation. Identification of tractable biological and psychosocial characteristics may allow caregivers to better highlight modifiable personal and contextual factors that impact functional outcomes and leverage the PWD’s retained assets to enhance mealtime participation.

Patient-centered treatment requires consideration of both tractable biological and psychosocial characteristics when managing feeding and swallowing impairments in PWD to ensure that the mealtime is as successful as possible. Central to a biopsychosocial approach is patient-centered, as opposed to, disease-centered care. For example, consider a patient with dementia in an isolated room who is exhibiting heightened levels of agitation. As they throw their lunch tray off the table, is this truly an example of BPSD? Or perhaps the patient is full, and the caregiver did not recognize cues to stop feeding? The caregiver’s response to this behavior may depend on the caregiver’s view of the behavior [24]. Coming from a disease-based perspective, the caregiver may view these behaviors as a result of the neurodegenerative disease process and disregard this attempted communication, potentially resulting in increased frustration for both the caregiver and the PWD [25]. Alternatively, by utilizing a person-centered approach, the caregiver can better recognize this behavior as a reaction to the interplay between biological, social, and environmental factors resulting in an attempt to communicate an unmet need [20,26]. By utilizing a person-centered perspective, the PWD’s needs may be better met, thereby alleviating further frustration for both the PWD and the caregiver. It is crucial for caregivers to look at how these individuals are attempting communication, both with verbal cues and non-verbal behavior. To view the patient and their retained abilities holistically, clinicians must consider how PWD are framed within the context of a degenerative disease. By combining aspects of the three theoretical models described above (WHO-ICF, Bisogni’s conceptual model of mealtime management, and the tractable characteristics of the Spector-Orrell model biopsychosocial model of dementia management) caregivers can utilize a person-centered biopsychosocial model of mealtime management for PWD that views patients’ actions as a result of not only the disease, but also the social and environmental processes.

## 3. A Biopsychosocial Model of Mealtime Management in PWD

**Figure 2:**
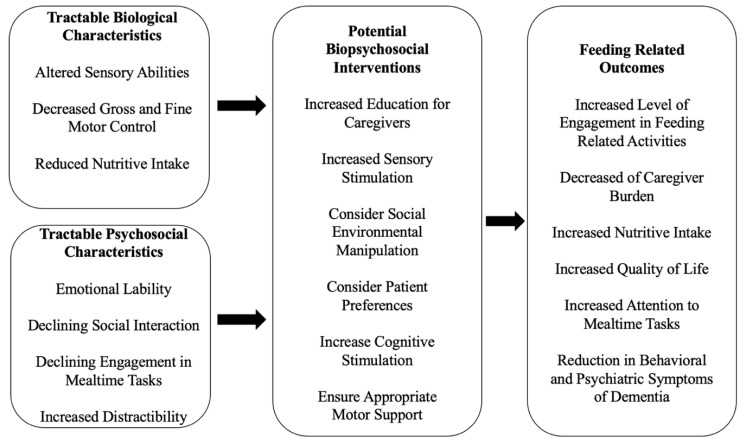
Dysphagia management in PWD utilizing a biopsychosocial model as an asset-based approach to patient-centered care.

Drawing on the previous theoretical models, in the remainder of this paper we introduce a biopsychosocial model of dysphagia management in PWD that utilizes an asset-based framework to identify how caregivers can better meet the needs of PWD and enhance their mealtime potential by capitalizing on the PWD’s retained capabilities. Such a model can allow caregivers to identify these retained capabilities, and leverage these capabilities for more successful mealtimes, as opposed to compensating for the disabilities associated with dementia. Figure 2 illustrates tractable characteristics commonly seen in PWD across two domains, biological and psychosocial. Addressing both domains allows for exploration of mealtime management with a focus on promoting success in mealtimes as opposed to mitigating the effects of mealtime breakdown. In addition to addressing the biological and the psychosocial processes often seen in PWD, this model introduces dysphagia management interventions that target both domains and explores feeding related outcomes. The model described below focuses on the tractable characteristics seen in PWD as these are the characteristics that caregivers can identify and manage as areas for intervention. Of note, these are commonly seen features around mealtime management in PWD and are commonly represented in the literature.

### 3.1. Tractable Characteristics

The left boxes in the model describe the tractable, or modifiable, biological and psychosocial changes that occur as a consequence of dementia. These characteristics are important to delineate given their potentially negative impact on the mealtime experience and the opportunity they provide for intervention.

#### 3.1.1. Tractable Biological Characteristics

The top left box in this model describes modifiable biological changes that can be addressed to best support mealtime management in PWD throughout the progression of their disease. Tractable biological characteristics related to mealtime management in PWD are varied and, if not addressed, may result in innumerable downstream deficiencies. Risk for nutrition deficiencies begin in the early stages of dementia with changes in the sensory system including reduced gustatory and olfactory senses [27]. Due to changes in motor control, PWD face reduced ability to participate in activities of daily living (ADLs), such as feeding and other mealtime related tasks, which may be a result of reduced fine motor control commonly seen as the disease progresses [28]. Changes in gross motor control may be a result of paratonic rigidity seen in dementia which can place the patient at increased risk of postural difficulties and reduced oropharyngeal swallow control [29,30]. Combined with other biological and psychosocial features related to cognitive decline, malnutrition is a significant risk for PWD [9,12].

#### 3.1.2. Tractable Psychosocial Characteristics

The bottom left box of the proposed model provides modifiable psychosocial changes that can be addressed to help support mealtime management in PWD. Tractable psychosocial characteristics related to mealtime management include the psychological changes seen in PWD and the way their social environments shape their participation in ADLs. Emotional and behavioral dysregulation have long been an identified as characteristics associated with emotional lability across many subtypes of dementia [31]. Identifying and effectively addressing emotional lability in PWD may be a crucial component in promoting mealtime engagement. Altered social interaction is a result of a confluence of factors associated with the dementias including altered cognitive status, communicative ability, or changes in sensory and motor ability [32,33,34]. The ability to engage in mealtime tasks is another psychosocial characteristic that may be altered in PWD, and successful mealtime engagement results from a variety of factors. Mealtime engagement can come in the form of direct attention from caregivers, social engagement with others who are participating in the mealtime, as well as engagement in tasks related to mealtime preparation [24,35,36,37]. Altered ability to socially participate across a wide variety of tasks is a known correlate of dementia progression [38,39], which may put PWD at increased risk of disengagement during mealtimes. Although caregiver preparedness is not a direct psychosocial domain of the person with dementia, it is included here because the caregiver directly manipulates the environment in which the PWD is receiving care.

### 3.2. Biopsychosocial Interventions and Outcomes

The middle box of the proposed model provides potential interventions that have been described previously in the literature. The righthand box provides associated mealtime outcomes that were described in the literature reviewed to create this model and suggest an improved mealtime experience for PWD.

#### 3.2.1. Caregiver Education

Caregiver education, as well as accessibility of caregiver education, is an important component when designing appropriate care for PWD. Availability of education is crucial for caregivers to meet the changing needs of PWD; however, there currently exists minimal to no standardized nutrition education for community-dwelling PWD and their caregivers [40]. Informal caregivers have expressed need for the availability of nutrition-based education in four main domains: meal preparation/food choices; addressing the PWD’s lack of appetite and altered eating behaviors; interpreting and synthesizing existing nutrition information; and identifying reliable nutrition information [40]. Through consistent education addressing the changing nutritional needs and capabilities of PWD, healthcare professionals may play an integral role in alleviating caregiver burden that is associated with lack of education surrounding mealtime management [41].

Implementation of a multicomponent caregiver education program addressing modifiable/contextual factors to decrease agitation in PWD can lead to decreased negative caregiver response, increased caregiver confidence, and improved patient outcomes [42]. Participation in educational programs by both formal and informal caregivers and psychological interventions geared at caregivers’ mindfulness has been shown to lead to a reduction in caregiver burden and an improvement in both QoL and depression scores for the caregiver and PWD [43,44]. Additionally, previous research has found that when individuals dine in a common room with a caregiver who has been trained to engage appropriately with PWD, there is an increase in dietary intake by 20% of total volume, which offers increased opportunity for weight gain and monetary savings as compared to nutritional supplements that are a costly alternative to food intake [45]. Reduced depression and burden as well as improved QoL could position caregivers to provide higher levels of care for the PWD while ensuring the health and wellbeing of the caregiver is maintained.

#### 3.2.2. Sensory Stimulation

The risk for nutritional deficiencies begins in the early stages of dementia alongside early changes in the sensory system, including reduced gustatory and olfactory senses [27]. Meeting the needs of the PWD is an evolving process as the disease progresses and requires scaffolding support during feeding activities. By utilizing an asset-based approach, caregivers can support PWD to participate in mealtimes despite changes in sensory functioning [24]. The level of scaffolding support should maximize retained strengths and capabilities of the PWD and will look different dependent on the progression of the disease process.

Tactile support can be provided in the form of hand feeding assistance. Individuals with advanced dementia may benefit from feeding assistance via a hand-under-hand presentation [46]. Hand-under-hand feeding assistance utilizes tactile cues from the caregiver and guides the PWD’s hand and utensil from the plate to the mouth. Additionally, utilizing hand-under-hand as a feeding assistance technique allows the PWD greater control over the direction and speed of movement, which may allow for greater feelings of autonomy [46,47,48]. The hand-under-hand feeding technique has been shown to reduce BPSD and increase intake, likely related, at least in part, to the PWD utilizing the preserved ability to provide cues for force and speed of movement [46,47]. Individuals with moderate dementia may not need tactile cues and may instead benefit from auditory cueing, with an emphasis on procedural repetition (e.g., “follow each bite with a drink”). Individuals with mild dementia may benefit most from goal-directed activities that increase sensory awareness of mealtimes (e.g., assisting in basic meal preparation) [24]. Supported meal-centric activities increase engagement of both the caregiver and the PWD while promoting increased sense of self, belonging, and identity during progression of the disease [24].

Auditory stimulation through the introduction of relaxing music played during mealtimes has been found to both decrease adverse mealtime behaviors while simultaneously increasing caloric intake in PWD [49,50]. Visual stimulation is known to play a role in caloric intake and weight management in PWD as well. PWD who were provided increased visual stimulation through the use of an aquarium placed in the dining area increased their weight by 2.2 pounds, on average, in a period of ten weeks [51]. Simple visual enhancement, such as increasing the contrast of crockery, has also been shown to increase intake of both food and liquid in PWD [52]. Increasing olfactory stimulation by infusing dining rooms with food smells prior to mealtime is another way sensory stimulation can lead to increased caloric intake in PWD [53]. Increasing sensory stimulation in PWD is a simple way to increase mealtime success through increased nutritive intake and decreased instances of BPSD. Yet, sensory stimulation is only one portion of the environment that caregivers can manipulate to increase mealtime success.

#### 3.2.3. Social Environment Manipulation

The environment in which a mealtime is taking place consists of a dynamic interplay between socialization and sensory stimulation. Environmental manipulation should extend beyond the physical environment to the social environment as well, which includes the individuals in the dining vicinity and the interactions that they have with the PWD [37,54]. Fostering a social dining environment that supports successful mealtimes may lead to increased nutritive intake [37]. Although not all PWD may display overt social interaction, utilizing visual cues from mealtime partners may encourage healthy food consumption for individuals that are less likely to participate in mealtimes by modeling appropriate mealtime behaviors [55]. Despite potential reductions in social interaction, for some PWD, when they eat in the presence of others who are also eating, they tend to increase the quantity of their intake [56].

Designing a mealtime environment that is conducive to nutritive intake is a multifaceted process, even more so in a dining room where there are multiple individuals engaging in mealtimes simultaneously. In addition to increasing nutritive intake, designing a mealtime environment that allows for socialization can lead to increased quality of life for PWD. By providing meals in a family style manner that allows for self-serving and socialization during mealtimes, PWD have reported increased quality of life, display increased fine motor functioning, and experience increased body weight [57]. These results support utilizing person-centered, asset-based principles of patient care by allowing the PWD to capitalize on their retained ability to serve themselves may increase intake as well as improve autonomy during mealtime tasks.

Although dementia is, by its progressive nature, a disease that results in an ever-changing set of symptomologies, PWD also benefit from consistency during mealtimes [39]. Maintaining a consistent and recognizable mealtime environment allows for the reduction of anxiety and distraction and can decrease cognitive load, allowing for increased attention to the mealtime task and increased nutritive intake [39].

#### 3.2.4. Patient Preferences

Sensory stimulation and social environment manipulation clearly have potential to increase nutritive intake, decrease BPSD, and increase QoL in PWD. However, for person-centered care to be truly person-centered, patient preference must be a salient feature of a biopsychosocial model of mealtime management. Unfortunately, discovering and honoring the preferences of a PWD can be difficult in light of the cognitive decline that accompanies a dementia diagnosis [58]. Due to the difficulty some PWD have with communicating their preferences, the responsibility to interpret attempts at communication falls to the caregiver. Despite communication difficulties, honoring the preferences of someone who is dependent on mealtime assistance by individualizing the mealtime experience is central to enhancing mealtimes [59]. The traditional biomedical model often results in PWD becoming passive participants in their care, whereas allowing opportunities for choice aligns with a biopsychosocial model of mealtime management. In order to make a choice, however, the PWD must have the capacity to choose [60]. Certainly, as cognitive decline progresses, the complexity of choice may need to be scaffolded to meet the abilities of the PWD. In the early stages of cognitive decline, a PWD may be able to verbalize their choice from a menu of options. Conversely, PWD with more severe cognitive decline may point to a desired food item or assert choice simply by closing their mouth to a food they are not interested in. In order to individualize the mealtime experience, caregivers may consider providing increased food variety [61]. Providing greater variety of flavor, texture, temperature, and quantity during mealtimes, and increasing frequency of mealtimes offered, may allow the PWD to have a greater ability to choose what and how they would like to eat, thereby increasing mealtime autonomy and the likelihood of consumption. Ultimately, caregivers can utilize an asset-based biopsychosocial model to support person-centered care by providing multiple options, honoring preferences of PWD, and aiming to increase mealtime autonomy.

#### 3.2.5. Cognitive Stimulation

Although it is well established that cognitive decline is the hallmark symptom of dementia, PWD may be able to learn and/or regain some level of cognitive functioning in order to better participate in mealtimes [62,63]. One method which has been shown to increase learning and retention of behavior in PWD is spaced retrieval [64]. Spaced retrieval is a method of learning that requires the individual to recall newly learned information over progressively longer periods of time and has been shown to increase learning and retention of behavior in PWD [64]. With use of this technique, PWD demonstrated significantly increased mealtime independence following training to recognize when mealtime was, masticate effectively, and generally required less assistance from caregivers during mealtimes [63].

Montessori-based programs that focus on breakdown of tasks, guided repetition, sequencing, and provision of feedback from caregivers have also been used to increase cognitive stimulation to facilitate learning of new skills in PWD [65]. Utilization of Montessori principles for mealtime management has been shown to lead to improvements in procedural skills needed for independent eating such as pouring, hand-eye-coordination, scooping, and discrimination of edible versus non-edible items [66]. Montessori-based principals have also been applied specifically to enhance person-centered mealtime practices by encouraging PWD to make more choices related to menu selections, increasing engagement, and increasing socialization during mealtime activities [67].

Increased engagement in mealtimes may also be achieved through practices that enhance attention to the mealtime task. Participation in a mindfulness program has demonstrated improvements in cognitive control, attention, and task switching tasks in PWD [44]. Mindfulness exercises that encourage attention to breathing, bodily sensations, body movement, and acceptance of thoughts have been shown to increase QoL, decrease depressive symptoms, and increase recall in PWD [44]. Improved cognitive and psychological performance may be seen in mealtimes for PWD following participation in mindfulness training; however, the potential mealtime benefits remain unknown and are currently under investigation [68].

#### 3.2.6. Adaptive Motor Support

Successful eating and swallowing requires appropriate cognitive, sensory, and motor functioning [69]. The process of eating and swallowing is often affected in PWD due to changes in cognition, sensory abilities and motor control in this population [10]. Functional upper-limb motor skills are required for effective self-feeding. Unfortunately, PWD often experience changes in upper-limb functioning [70]. Moreover, a decline in upper-limb functioning is associated with reduced ability to participate in ADL’s such as eating and dressing thereby reducing autosnomy [71,72]. In part due to this loss of autonomy and reduced motor ability, PWD often experience reduction in food intake and have poor nutritional status [73]. Management of motor changes in PWD has been addressed broadly in two categories, compensatory and rehabilitative.

Acquisition, as well as long-term retention, of fine motor skills in PWD have been found through various means of training. Learning of fine motor hand movements that require hand-eye coordination have been found in PWD following repeated practice with a rotary pursuit task [74]. The rotary pursuit task involves using a pointer to follow a moving target across a screen. As applied to mealtimes, the finding that such a skill could be acquired even by individuals with severe dementia raises the possibility that repeated practice of functional skills (e.g., movement of utensil from plate to mouth) may be used to increase fine motor skills in PWD during mealtimes. Interestingly, acquisition of one fine-motor skill through the use of continuous repeated practice has also been shown to transfer to non-practiced fine motor movements in PWD [75]. Transfer of fine motor ability from practiced to non-practiced tasks further supports that continuous repeated practice of functional motor skills may increase ability to participate in mealtimes. Acquisition of new skills, however, requires training of these skills, which may not be possible given the myriad responsibilities burdening caregivers of PWD.

When acquisition of new skills is not a feasible intervention, compensatory strategies may be more appropriate to support retained motor abilities in PWD. In addition to providing additional sensory stimulation as discussed previously, hand-over-hand feeding techniques have been utilized to support changes in the motor control in PWD as well (Batchelor-Murphy, 2016; Batchelor-Murphy et al., 2017). Another widely used compensatory strategy to address swallowing difficulties due to changes in motor control is the modification of food textures [76,77]. However, there is conflicting support demonstrating the effectiveness of texture modified food on safe intake [78]. Utilization of nutrient-enhanced between meal supplements has been shown to increase nutrient intake and weight in both nursing home residents as well as PWD [79,80].

Changes in cognitive functioning and motor control may result in PWD displaying difficulty with utensil use as well as difficulty with transit of food from plate to mouth [81]. Interventions investigating the use of foods that are easily picked up, or “finger foods”, have sought to circumvent the use of utensils. The use of finger foods has been shown to increase intake of fruits and vegetables in PWD when compared to provision meals that do not include finger foods [82]. In addition to being easier to pick up and transport to the mouth, finger foods may provide an additional opportunity for increased tactile and visual stimulation. Similar to findings that high visual contrast crockery leads to increased nutritive intake, finger foods that were of high visual contrast were consumed in a larger quantity than finger foods with low visual contrast [83]. Pouyet et al. (2014) additionally investigated choice in PWD by providing a sauce as an additional option to the finger foods. PWD demonstrated significantly increased choice of the finger food with the provision of a sauce versus finger foods provided alone [83]. Ultimately, both compensatory and rehabilitative interventions demonstrate the ability to capitalize on the retained motor assets of PWD despite changes in motor function in order to increase nutritive intake.

## 4. Discussion

Successfully assisted mealtimes are a critical component of caring for PWD as mealtimes have large impacts on QoL, maintenance of nutrition, feelings of autonomy, and socialization [12,15,16,73,84,85,86]. Additionally, considerations for mealtime management must reflect the dynamic relationship between the biological and psychosocial characteristics that impact the ability of PWD to participate in mealtimes. Biopsychosocial interventions that utilize an asset-based approach to mealtime management have been shown to increase nutritive intake, increase QoL, and decrease BPSD in PWD [44,45,56,67,75,83].

The Biopsychosocial Model of Mealtime Management proposed here may be a crucial component in designing person-centered mealtime interventions. However, in order to create meaningful, functional treatment options for PWD, future research in the area of dietary management must consider the perspectives of PWD. While it is understood that a progressive neurological disease will change the way an individual communicates, research has shown that PWD retain the ability to communicate their choices and opinions regarding mealtime management [85]. Unfortunately, however, many PWD report feelings of loss of agency and control when they are not provided opportunities to make choices [85]. Given the importance that recognition of choice and preference has on mealtime participation and maintenance of nutrition [67,83], it is crucial that future research surrounding mealtime management considers the perspectives of PWD. The capacity for an individual with decreased cognitive ability to participate in research may be difficult to ascertain, often resulting in clinical practices that position the PWD as a passive participant of intervention [87,88]. This “default view” needs to be re-examined. Future research must challenge this deficit-based perspective to identify areas where PWD can be integrated into research and clinical practice to better understand where mealtimes can be enhanced. The first step to ensuring truly person-centered care is to consider the perspective of, and minimize compromises to, the dignity of PWD [88]. Utilization of an asset-based biopsychosocial framework may guide the caregiver’s perspective away from an orientation that highlights areas of breakdown in mealtime and instead asks the caregiver to consider capitalizing on retained assets to promote successful mealtimes.

Mealtime management is a crucial component in considering care for PWD. The multifaceted nature of the mealtime experience for PWD, including increased reliance on caregivers, decreased opportunities for socialization, and changes in sensorimotor function, highlight the need for a dynamic approach to mealtime care. Moreover, the interconnected features of eating and drinking episodes suggest that single-component interventions, such as increasing the contrast of food or playing calming music, may not be enough to fully support PWD during mealtimes. Rather, as we propose here, successful mealtimes may be reliant on the consideration and explicit targeting of sensorimotor functioning, dining environment, cognitive status, and preferences of PWD as integral parts of mealtime management. PWD should remain active participants in their care. Utilizing an asset-based biopsychosocial model of mealtime management, that requires centering on the unique strengths and capabilities of each PWD, yields the potential to improve a broad range of mealtime outcomes more effectively, including mealtime engagement, autonomy, and nutrition maintenance.
